# Randomized controlled multicenter trial on the effectiveness of the collagen hemostat Sangustop^®^ compared with a carrier-bound fibrin sealant during liver resection (ESSCALIVER study, NCT00918619)

**DOI:** 10.1007/s00423-014-1203-9

**Published:** 2014-06-01

**Authors:** C. Moench, A. L. Mihaljevic, V. Hermanutz, W. E. Thasler, K. Suna, M. K. Diener, D. Seehofer, H. J. Mischinger, B. Jansen-Winkeln, H. P. Knaebel, W. O. Bechstein

**Affiliations:** 1 Department of General and Visceral Surgery, Goethe-University Hospital and Clinics, Theodor Stern Kai 7, 60590 Frankfurt am Main, Germany; 2 Department of Surgery, Klinikum rechts der Isar, Technische Universität München, Munich, Germany; 3 Aesculap AG, Tuttlingen, Germany; 4 Department of General, Visceral, Transplantation, Vascular and Thorax Surgery, Grosshadern Hospital, Ludwig Maximilians University, Munich, Germany; 5 Department of Surgery, Krankenhaus Nordwest, Frankfurt am Main, Germany; 6 Department of General, Visceral and Transplantation Surgery, University of Heidelberg, Heidelberg, Germany; 7 Department of General, Visceral and Transplantation Surgery, University Hospital Berlin - Charité, Berlin, Germany; 8 Department of Surgery, Medical University Graz, Graz, Austria; 9 Department for General, Visceral and Transplantation Surgery, University of Mainz, Mainz, Germany

**Keywords:** Randomized controlled trial, Liver resection, Carrier-bound fibrin sealant, Collagen hemostat, Hemostatics

## Abstract

**Background:**

Despite improvements in liver surgery over the past decades, hemostasis during hepatic resections remains challenging. This multicenter randomized study compares the hemostatic effect of a collagen hemostat vs. a carrier-bound fibrin sealant after hepatic resection.

**Methods:**

Patients scheduled for elective liver resection were randomized intraoperatively to receive either the collagen hemostat (COLL) or the carrier-bound fibrin sealant (CBFS) for secondary hemostasis. The primary endpoint was the proportion of patients with hemostasis after 3 min. Secondary parameters were the proportions of patients with hemostasis after 5 and 10 min, the total time to hemostasis, and the complication rates during a 3 months follow-up period.

**Results:**

A total of 128 patients were included. In the COLL group, 53 out of 61 patients (86.9 %) achieved complete hemostasis within 3 min after application of the hemostat compared to 52 out of 65 patients (80.0 %) in the CBFS group. The 95 % confidence interval for this difference [−6.0 %, 19.8 %] does not include the lower noninferiority margin (−10 %). Thus, the COLL treatment can be regarded as noninferior to the comparator. The proportions of patients with hemostasis after 3, 5, and 10 min were not significantly different between the two study arms. Postoperative mortality and morbidity were similar in both treatment groups.

**Conclusion:**

The collagen hemostat is as effective as the carrier-bound fibrin sealant in obtaining secondary hemostasis during liver resection with a comparable complication rate.

**Electronic supplementary material:**

The online version of this article (doi:10.1007/s00423-014-1203-9) contains supplementary material, which is available to authorized users.

## Introduction

Improvements in preoperative imaging, perioperative care and surgical technique have led to expansion of hepatic surgery during the last decades including extended hepatic resections [1, 2]. Likewise, the number of hepatic resections has increased [3, 4], while perioperative outcome has improved [3–5]. Nonetheless, liver surgery is still associated with substantial mortality and morbidity rates even at high volume centers [6]. In addition, the advent of neoadjuvant chemotherapy, especially in patients with colorectal liver metastases [7], demographic changes in Europe and the USA with an increasing number of elderly patients as well as the rising number of liver resections in patients with impaired liver function have increased the likelihood of complications in hepatic surgery. Therefore, safe liver surgery remains challenging and a main objective not only for surgeons and patients but also for health care providers, since complications following liver surgery are associated with substantial health care costs [8, 9].

A frequent contributor to intraoperative complications is hemorrhage from the resection surface which occurs independent of the resection technique and is frequently triggered by impaired coagulation. Furthermore, hematoma as well as bile leakage from the resection surface can lead to troublesome postoperative complications. Therefore, rapid and effective treatment of bleeding during and after hepatic surgery reduces blood loss and may help to reduce perioperative complications. It subsequently may reduce the need for transfusion, operative time, and duration of hospital stay.

In order to achieve control over parenchymal diffuse bleeding and complications attributed to bleeding, various locally applicable hemostatic agents are in use. These agents include bone wax, gelatin, collagen, oxidized cellulose, fibrin sealants glues, and synthetic glues [10, 11]. Some evidence from randomized controlled trials (RCTs) exists regarding the use of fibrin sealants on their own [12–14] or combined with a carrier matrix [15–18]. However, the routine use of fibrin sealants has to be judged against noticeable additional costs. A combined product with well-documented efficacy is Tachosil^®^. It consists of a collagen patch carrying the fibrin glue components human fibrinogen and human thrombin. It was shown in two RCTs [16, 17] to be superior in obtaining intraoperative hemostasis over argon plasma coagulation in liver resection by reducing the time to hemostasis significantly.

The collagen fleece Sangustop^®^ is a novel means of treating regional bleeding during surgical procedures. It is made up of a mesh-like matrix of absorbable collagen fibrils to ensure flexibility of the hemostat while at the same time preserving tensile strength. The matrix allows for an increased surface area to enhance the hemostatic effect. In contrast to the carrier-bound fibrin sealant, it does not contain any pharmacologically active components. The objective of this randomized controlled multicenter trial was to assess if the collagen hemostat Sangustop^®^ is as effective as the carrier-bound fibrin sealant Tachosil^®^ with regard to hemostatic efficacy and safety.

## Materials and methods

### Design

The study was designed as a prospective, noninferiority, multicenter, two-arm, randomized, single-blinded study. To show transparency the protocol of the *E*fficacy and *S*afety of *S*angustop^®^ as hemostatic agent vs. a *CA*rrier-bound fibrin sealant during *LIVER* resection (ESSCALIVER) study was registered at http://www.clinicaltrials.gov (identifier: NCT00918619), and the study protocol was published previously [19]. Ethical approval was obtained from the responsible ethics committees. The study was sponsored by Aesculap AG (Tuttlingen, Germany). Clinical monitoring and data management were contracted to Centrial GmbH (Tübingen, Germany). Statistical planning and analysis was performed by Dr. M. Koehler GmbH (Freiburg, Germany).

### Participants

Only patients aged over 18 years scheduled for an elective, open liver resection (segmental or nonsegmental) were eligible for participation in the study. Exclusion criteria were presence or sequelae of coagulation disorders, medical history or clinical evidence of liver cirrhosis, Klatskin tumor, participation in another clinical study within the last 30 days, pregnancy or breast feeding, concurrent or previous therapy with systemic pharmacologic agents promoting blood clotting (including but not limited to tranexamic acid, activated factor VII, and aprotinine), and known allergy or hypersensitivity to human thrombin or to human fibrinogen or to riboflavin or to proteins of bovine origin. Furthermore, a number of intraoperative exclusion criteria, evaluated immediately after liver resection, had to be ruled out before intraoperative randomization could be carried out, namely, resection area estimated by operating surgeon to be less than 16 cm^2^, an infected wound area, and persistent major bleeding or no bleeding after primary operative hemostatic procedures.

Written informed consent was obtained from all eligible patients during the screening visit.

### Surgical technique and intervention

The liver surgery was performed according to the respective center’s local standards. The only provisions were regarding the dissection techniques and the primary hemostatic methods. The following techniques of liver resection were allowed: Cavitron Ultrasonic Surgical Aspirator (CUSA^®^), Hydrojet^®^, clamp crushing, scissors, and stapler transection. Any techniques of liver resection with a coagulation activity were not permitted, e.g., argon plasma coagulation (APC), Habib^™^-Sealer, Tissue Link^®^, and UltraCision^®^.

For achieving primary hemostasis, only sutures and vascular clips were permitted, and it was performed in accordance with the preferences of the surgeon. If complete hemostasis was achieved with these primary hemostatic measures (i.e., no bleeding from the resection area), then the patient had to be excluded from the trial. In case of persisting bleeding from the resection area, adequate surface size patients were randomized to either the COLL or the CBFS group.

#### COLL group

Sangustop^®^ fleeces (Aesculap AG) measuring 5 × 8 cm were applied to the bleeding resection area. Mild pressure was applied using dry instruments until the collagen fleece adhered to the resection surface. The resection area had to be covered completely with the fleece, and an overlap of >1 cm was mandatory in cases were more than one product was used. Additional fleeces could be put on the site at any time during the measurement phase if blood seeped through the fleece. The fleeces remained in situ as they are bioabsorbable. Upon application of the collagen hemostat to the resection surface, a stopwatch was started and the bleeding site was inspected every minute from the beginning until bleeding stopped, for a maximum of 10 min.

#### CBFS group

Tachosil^®^ (Nycomed, Linz, Austria) patches measuring 4.8 × 9.5 cm were applied to the resection area after being moistened with saline solution. The yellow-coated side of the patch containing the active hemostatic components was applied against the resection surface for a minimum of 3 min according to the manufacturer’s instruction. After this period, hemostasis was controlled for the first time, followed by inspections after every minute until bleeding was stopped, but for a maximum of 10 min. As for the COLL group, the resection area had to be covered completely with a mandatory overlap of >1 cm in cases where more than one patch was used. Additional patches could be used at any time during the measurement phase if blood seeped through the patch. The patches remained in situ as they are bioabsorbable.

Hemostasis was defined as being achieved when there was no visible bleeding from the target site, neither through nor from the sides of the fleeces/patches. Evaluation was done by the operating surgeon/investigator. If hemostasis was not achieved within 10 min, other methods of hemostasis were allowed in both groups. No specific treatment was defined and all measures were allowed without restrictions.

After completion of the hepatectomy, a blot of the transection surface was made on a sheet of paper, and later on, this area was calculated from weighing the cutout piece of the surface area imprint. The results of the histopathology study with regard to the nontumorous associated liver microstructure (e.g., presence of steatosis, fibrosis, etc.) and the transfusion units given within the first week were recorded.

### Outcomes

The objective of the ESSCALIVER trial was to show that the COLL hemostat is noninferior to the CBFS in stopping bleeding after liver resection. The primary endpoint was complete hemostasis within 3 min after application of the hemostat. We choose this endpoint because the CBFS has to be pressed to the bleeding site for a minimum of 3 min according to the manufacturer’s instruction. Hence, 3 min was the earliest time point when hemostasis could be evaluated in both groups.

Secondary efficacy variables were the proportion of patients with hemostasis after 5 and 10 min as well as the time to hemostasis. To assess safety, the complication rate was documented in both treatment groups until 3 months postoperatively.

After 1 month (±5 days) and after 3 months (±10 days), patients were phoned by the investigator or study assistant. Using a predefined questionnaire, patients were asked open-ended questions to evaluate whether he/she had experienced any complications during the interim time interval.

### Randomization

A 1:1 intraoperative randomization was performed using identical looking, sealed, and numbered opaque envelopes. Randomization was stratified by study center. Lists with a block size of 4 were generated for each participating center prior to the initiation of the study using the Software RandList of the DatInf GmbH (Tübingen, Germany). Envelopes had to be opened in a sequential manner, after intraoperative inclusion criteria were met by one of the participating investigators. The eligible investigator who opened the randomization envelope had to date and sign the randomization sheet. The sequence of opening the envelopes was monitored regularly.

### Blinding

ESSCALIVER is a single-blinded trial, i.e., patients were not informed about their assignment in order to increase reliability of secondary outcomes, assessed during the follow-up visits. Due to the appearance of the products used and the differences in their application, blinding of the primary outcome assessor was not possible. However, since randomization was performed after the liver transection, the investigators performed the transection, the primary hemostasis, and the assessment of the intraoperative inclusion criteria without knowing which hemostatic product was going to be tested.

### Statistics

#### Primary variables

To show that COLL is noninferior to CBFS in regard to hemostasis, the following noninferiority design was chosen. Noninferiority was demonstrated if the lower limit of the observed two-sided 95 % confidence interval of the observed difference in proportions of treatment arm 1 (COLL)−treatment arm 2 (CBFS) does not fall below −0.100. The analysis was based on the per protocol population and repeated for the intent-to-treat population, since in a noninferiority trial use of the per protocol analysis set is generally seen as the conservative approach. If the 95 % confidence interval for the treatment effect not only lies entirely above –0.1 but also above zero, then there is evidence of superiority in terms of statistical significance at the 5 % level. The difference in proportions between the two treatment arms (treatment arm 1 vs. treatment arm 2) was tested with a Fisher’s exact test to reject the null hypothesis of no difference.

#### Secondary variables

The proportion of patients with hemostasis 5 and 10 min after application was reported descriptively based on the intent-to-treat population as well as the per protocol population. This includes the proportions, the estimated difference in proportions, and the associated 95 % confidence intervals. Differences in time to hemostasis were tested with a log–rank test at the 5 % alpha level. Kaplan–Meier curves were displayed, with median estimates and confidence limits provided. The analyses were based on the intent-to-treat population and repeated for the per protocol population. Adverse events (AEs) were summarized and categorized by severity (mild, moderate, and severe), seriousness (serious or nonserious), and relationship to device (certain, probable, possible, unlikely, and not assessable).

#### Sample size

The sample size was calculated to test for noninferiority of the control group vs. the experimental group in terms of hemostasis 3 min after application of the hemostatic product. In a previous study the proportion of patients with complete hemostasis after 3 min was estimated to be 73 % for the control [17]. With 60 subjects in each group, the lower limit of the observed one-sided 97.5 % confidence interval will be expected to exceed −0.100 with 93 % power when the proportion of the treatment arm 2 (CBFS) is 0.730 and the expected proportion of the treatment arm 1 (COLL) is 0.880. Results are based on 100 simulations using the Newcombe–Wilson score method to construct the confidence interval. Assuming a drop-out rate of 5 %, a total number of 126 patients are needed to be enrolled. The expected difference of 15 % was justified by preclinical data [20].

## Results

A total of 137 patients consented to participate between January 2010 and October 2010. Of those, 128 patients were available for intraoperative randomization. Reasons for drop-out before randomization were: dry resection area (4), irresectable tumor (3), resection area too small (1), and consent withdrawn (1). One patient was excluded because between intraoperative randomization and the application of the hemostats, the resection area was found to be dry. Thus, 127 patients (COLL: *n* = 62 and CBFS: *n* = 65) received a study intervention and were available for evaluation. In one patient, the investigational product COLL was applied wet instead of dry. This represented improper use of the product and the patient had to be removed from the per protocol analysis.

**Fig. 1:**
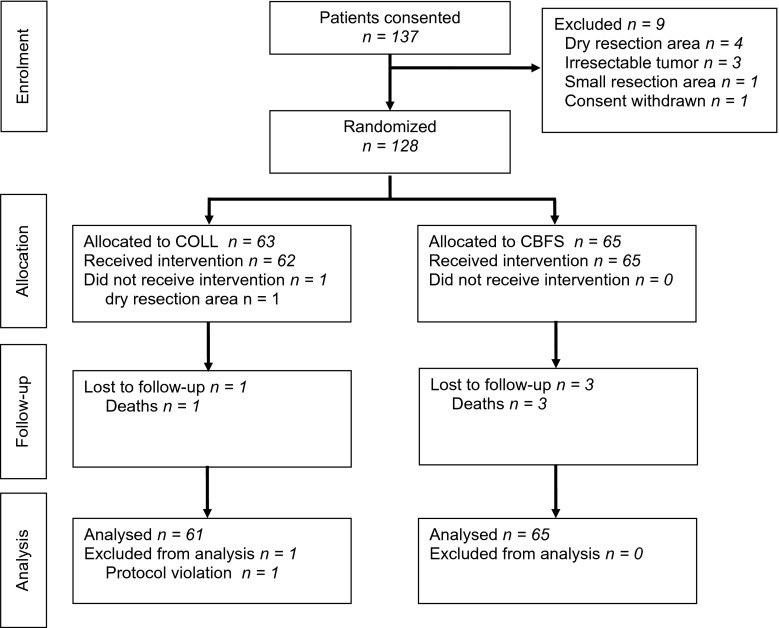
CONSORT flow chart

Patients were recruited in eight study centers (6 to 32 patients per center). The CONSORT-type diagram (Fig. 1) shows the flow of participants through each stage of the study.

The two study groups were comparable for all patients and procedural characteristics. Demographics and laboratory data at baseline are shown in Table 1. The data recorded during the surgical procedure are summarized in Table 2. The results of the histopathological examination on nontumorous liver microstructure is shown in Table 1. In the COLL group, 53 out of 61 patients (86.9 %) reached hemostasis 3 min after application of the product compared to 52 out of 65 patients (80.0 %) in the CBFS group. The 95 % confidence interval for the difference of 6.9 % was [−6.0 %, 19.8 %]. Since the lower limit was above −10 %, the COLL treatment is regarded as clinically noninferior to the comparator CBFS treatment. In addition a statistical comparison of the devices was performed using Fisher’s exact test. The two-sided test showed a *p* value of 0.3453.

**Table 1:** Patient demographics

	COLL (*n* = 62)	CBFS (*n* = 65)
Demographics		
Male, *n* (percent)	34 (55 %)	40 (62 %)
Mean age years, mean (SD)	61.0 (12.8)	61.9 (13.2)
Body mass index, mean (SD)	26.6 (4.2)	28.1 (14.3)
ASA status		
P1*, n* (percent)	13 (21 %)	9 (14 %)
P2	31 (50 %)	33 (51 %)
P3	18 (29 %)	23 (35 %)
Preoperative chemotherapy	21 (34 %)	24 (37 %)
Underlying diagnosis		
Liver metastasis	38 (61 %)	42 (65 %)
Hepatocellular carcinoma	5 (8 %)	11 (17 %)
Adenoma of the liver	3 (5 %)	3 (5 %)
Cholangiocellular carcinoma	2 (3 %)	3 (5 %)
Gall bladder carcinoma	4 (6 %)	1 (2 %)
Hemangioma	2 (3 %)	2 (3 %)
Carcinoma of unknown primary origin	2 (3 %)	2 (3 %)
Focal nodular hyperplasia (FNH)	1 (2 %)	–
Neuroendocrine tumor (NET)	1 (2 %)	–
Others	4 (6 %)	1 (2 %)
Histopathology		
Steatosis	18 (29 %)	17 (26 %)
Fibrosis	13 (21 %)	14 (22 %)
Steatohepatitis	2 (3 %)	1 (2 %)
Baseline laboratory data		
International normalized ratio, mean (SD)	1.023 (0.1034)	1.020 (0.0937)
Partial thromboplastin time in second, mean (SD)	31.16 (4.724)	30.80 (5.930)
Hemoglobin in gram per deciliter, mean (SD)	13.12 (1.639)	13.60 (1.436)
Hematocrit in percent, mean (SD)	39.36 (3.981)	40.51 (3.667)

**Table 2:** Intraoperative data according to surgical intervention

	COLL (*n* = 62)	CBFS (*n* = 65)
Techniques of liver resection		
CUSA^®^	37 (60 %)	37 (57 %)
Scissors	22 (35 %)	19 (29 %)
Clamp crushing	13 (21 %)	15 (23 %)
Stapler	9 (15 %)	14 (22 %)
Hydrojet^®^	1 (2 %)	1 (2 %)
Method of primary hemostasis		
Sutures	57 (92 %)	58 (89 %)
Clips	51 (82 %)	52 (80 %)
Resection		
Segmental	43 (72 %)	51 (80 %)
Nonsegmental	17 (28 %)	13 (20 %)
Segmental and nonsegmental	2 (3 %)	1 (2 %)
Weight of resected liver in grams, mean (SD)	414 (357)	386 (302)
Resection area in square centimeters, mean (SD)		
Estimated by surgeon	81 (65)	66 (42)
Measured from blot	83 (51)	79 (41)
Central venous pressure in millimeters of mercury, mean (SD)	6.7 (4.8)	5.9 (4.9)
Pringle yes, *n* (percent)	24 (39 %)	28 (43 %)

**Fig. 2:**
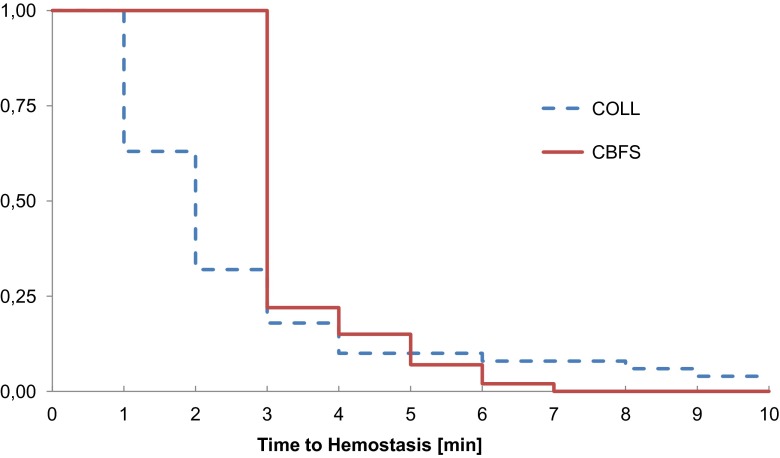
Kaplan–Meier curves for the time to hemostasis for COLL (*dashed line*) and CBFS

The proportion of patients with hemostasis 5 min after application of the hemostatic products was 93 % for the COLL group and 95 % in the CBFS group, CI 95 % [−10.0 %, 6.1 %]; *p* = 0.7114. In one patient treated with COLL, hemostasis was not achieved after 10 min and additional sutures were applied after that time. In the CBFS arm, hemostasis was achieved in all patients within 10 min. In total, 98 % of patients receiving the COLL hemostat and 100 % of patients treated with CBFS obtained hemostasis within 10 min, with CI 95 % [−4.8 %, 1.6 %]. The mean time to hemostasis was calculated to be 2.2 (SD ± 1.60) min for the COLL arm and 3.38 (SD ± 0.88) min for the CBFS arm. Note that due to the different mode of application, the time measurement started at time 0 min in the COLL arm and at 3 min for the CBFS arm. Thus, the two cannot be compared with each other within the first 3 min. The Kaplan–Meyer curves are shown in Fig. 2. The repeat of the analysis of primary and secondary efficacy variables using the ITT population showed similar results and confirmed the finding of the noninferiority of the COLL treatment vs. the comparator CBFS.

A total of 152 COLL fleeces were used in 62 patients (mean 2.45; minimum one piece and maximum seven pieces per patient). A total of 151 CBFS patches were applied in 62 patients (mean 2.32; minimum one piece and maximum four pieces per patient). The calculated use of products per cm^2^ resection area was similar in both treatment groups (COLL: 0.04 ± 0.022 and CBFS: 0.03 ± 0.016; *p* = 0.8263). The blood parameters were tested on the first postoperative day. They were within the normal values and did not differ between the two treatment groups. Blood transfusions were necessary in 20 COLL patients (33 %) and in 19 CBFS patients (29 %) during the first postoperative week.

### Safety analysis

Four patients died during the course of the study resulting in an overall 3-month mortality of 3.2 %: one patient in the COLL group (heart failure 8 days after surgery) and three patients in the CBFS group (myocardial infarction 14 days after surgery; multiorgan failure 41 days after surgery; multiorgan failure 28 days after surgery). None of the deaths was associated with the hemostatic agents. Furthermore, no hemostat-related adverse event was reported. Overall, 267 adverse events (AEs) were reported in 77 patients during the course of the study (121 AEs in 38 patients in the COLL group and 146 AEs in 39 patients in the CBFS group; Table 3). The most common AEs were anemia, constipation, and abdominal pain. A total of 21 complications were rated as serious in 16 patients (26 %) in the COLL group, whereas 29 serious complications occurred in 23 patients (35 %) in the CBFS group. The majority of serious complications were related to the surgical procedure, the underlying disease, or the preoperative health status. The most frequent serious surgical complications were biliomas (3.2 % in COLL and 4.6 % in CBFS), bile leaks (3.2 % in COLL and 3.1 % in CBFS), intraabdominal fluid collection (3.2 % in COLL and 3.1 % in CBFS), and wound infection or dehiscence (1.6 % in COLL and 4.6 % in CBFS; Table S4). There were no unplanned reoperations in our study, but one patient in the COLL group underwent planned relaparotomy within the study period for complete tumor resection.

**Table 3:** Adverse events

	COLL (*n* = 62)	CBFS (*n* = 65)
Total number of adverse events	121	146
Patients with at least one adverse event	38 (61.3 %)	39 (60.0 %)
Adverse events with an occurrence of >5 %		
Anemia	14 (22.6 %)	14 (21.5 %)
Constipation	7 (11.3 %)	7 (10.8 %)
Abdominal pain	5 (8.1 %)	6 (9.2 %)
Nausea	5 (8.1 %)	6 (9.2 %)
Seroma	3 (4.8 %)	4 (6.2 %)
Thrombocytopenia	4 (6.5 %)	3 (4.6 %)
Hypokalemia	4 (6.5 %)	3 (4.6 %)
Vomiting	4 (6.5 %)	2 (3.1 %)
Wound infection	2 (3.2 %)	4 (6.2 %)

## Discussion

Hemostasis in hepatic surgery is of considerable clinical significance due to the high number of liver resections performed each year, the significant morbidity and mortality [3, 4]) as well as the substantial health care costs [21] associated with intraoperative or postoperative hemorrhage in liver surgery. Furthermore, inadequate hemostasis during hepatectomy is associated with the unfavorable need for blood transfusion [22], postoperative complications [23, 24], increased operative time, and early tumor recurrence [25]. At the resection margins larger vessels and bile ducts are either clipped or ligated with sutures. However, parenchymal bleeding can still persist. Other methods for controlling this secondary bleeding are available, including fibrin glue, patches containing coagulation factors as well as collagen and cellulose based materials. In the current multicenter, randomized, controlled trial, we compared the efficacy and safety of a collagen fleece COLL without coagulation factors to a carrier-bound fibrin sealant CBFS for secondary hemostasis in elective open liver resections.

The study population was recruited from patients referred to the study centers for elective liver surgery. By applying wide inclusion and exclusion criteria, the Esscaliver trial aimed to reflect everyday clinical practice and intended to increase generalizability. Furthermore, since neither the surgical resection procedure nor resection instruments were standardized with few exceptions (markedly a minimum resection surface of 16 cm^2^ and the avoidance of argon plasma coagulation for primary hemostasis), the results of the trial seem to be applicable to wide range of hepatectomy procedures, indications, and surgeons.

Our results allow the conclusion that, given the predefined margins of noninferiority, the collagen hemostatic fleece can be regarded as noninferior to the comparator carrier-bound fibrin sealant in achieving secondary hemostasis in liver resections. Both treatment options were highly effective with 86.9 % of patients in the COLL arm and in 80.0 % of patients in the CBFS arm achieving complete hemostasis after 3 min. Furthermore, after 5 min and after 10 min, hemostasis was achieved in all but one patient, underlining the efficacy of both COLL and CBFS. The median time to hemostasis was reduced by about 1 min after the application of COLL (2.2 min) in comparison to CBFS (3.4 min). This difference is not necessarily due to a different hemostatic efficacy. It might also reflect specificities in trial design. The CBFS needs to be applied for a minimum of 3 min according to the manufacturer’s instruction, while the same limitation does not exist for COLL. Evaluation for complete hemostasis was censored till this time point for the CBFS but not the COLL group.

The validity of our results is underlined by comparing our data to previous trials that have employed a carrier-bound fibrin sealant and have found similar median hemostatic times. Fischer at al. [16] reported a median hemostatic time of 3.6 min and Frilling et al. [17] a median time of 3.9 min. This is very close to the reported 3.4 min observed in this trial. We could rule out the possibility that the equivalent hemostatic effect was due to an increase use of COLL as the mean number of fleeces/patches used per area was similar in both treatment arms.

In our study, the safety profile of both treatments seems to be comparable, evidenced by the almost identical percentage of patients experiencing an adverse event in both study arms, as well as the distribution of the most frequent adverse events. Furthermore, the number and frequency of patients requiring blood or blood components transfusions within the first postoperative week was very similar in both treatment arms indicating that there is no difference with regard to postoperative bleeding complications.

We rule out the possibility of a random effect on our results since the studies’ baseline characteristics such as age, gender, BMI, ASA status, baseline blood values, and indication for liver resection were well balanced. In addition, the intraoperative data regarding surgical technique and histopathological findings of nontumorous liver tissue were evenly distributed over the two study arms indicating that the randomization process was effective in producing comparable study groups differing only in the way of secondary hemostatic treatment.

A clear limitation of our trial was the restriction to high-volume surgical centers with high expertise in liver surgery, which might limit generalizability of the results. Furthermore, the single-blinded trial design with the inability to blind the primary outcome assessor was a limitation that might potentially bias the results of our trial. We have tried to minimize this effect by implementing an intraoperative randomization process that occurs only after key surgical steps (exploration, resection, and primary hemostasis) have taken place. Another limitation could be that the measurement of the time to hemostasis is a subjective assessment and prone to bias. However, currently, there seems to be no better method available, which is reflected by the fact that it is the method of choice in comparable studies [26, 27]. Furthermore, it is accepted by the FDA as a primary endpoint for pivotal studies of fibrin sealants [28].

Some other properties of the COLL have not been investigated specifically in this study but should be mentioned. Handling of the COLL is equivalent to the CBFS with one marked difference: the COLL fleece does not need a presoaking and can be used directly without activation, and thus, it can be applied laparoscopically too. The COLL fleece is absorbed more rapidly than the CBFS system (within 3 weeks vs. 12 weeks). Finally, although we did not perform a cost effectiveness analysis in our trial, increasing the options of effective hemostatic agents with comparable safety profile allows to consider economic aspects in the choice of hemostats.

## Conclusion

In summary, this controlled multicenter randomized study comparing a novel collagen hemostyptic fleece to an established carrier-bound fibrin sealant revealed the noninferiority of the collagen fleece in hepatic surgery. The results of this study increase the available options for surgeons and allows them to make choices based on surgical sites, handling characteristics, or cost-effectiveness.

## Electronic supplementary material

Below is the link to the electronic supplementary material.

Table S4 (PDF 15 kb)
