# Supplementary material for: Randomized controlled multicenter trial on the effectiveness of the collagen hemostat Sangustop® compared with a carrier-bound fibrin sealant during liver resection (ESSCALIVER study, NCT00918619)
Source: Langenbecks Arch Surg. 2014 Jun 1;399(6):725–33. doi: 10.1007/s00423-014-1203-9 (PMC4099526; doi:10.1007/s00423-014-1203-9)
Supplement: Supplementary file 1 — (PDF 15 kb) [file 423_2014_1203_MOESM1_ESM.pdf]

Supplement Table 4

***Table 4*: Most frequent serious surgical complications**

	COLL<br>n = 62	CBFS<br>n = 65
Bilioma	2 (3.2%)	3 (4.6%)
Bile leak	2 (3.2%)	2 (3.1%)
Inraabdominal fluid collection	2 (3.2%)	2 (3.1%)
Impaired wound healing (infections, dehiscence)	1 (1.6%)	3 (4.6%)
Post procedural haemorrhage	1 (1.6%)	1 (1.5%)
